# Pneumolysin promotes host cell necroptosis and bacterial competence during pneumococcal meningitis as shown by whole-animal dual RNA-seq

**DOI:** 10.1016/j.celrep.2022.111851

**Published:** 2022-12-20

**Authors:** Kin Ki Jim, Rieza Aprianto, Rutger Koning, Arnau Domenech, Jun Kurushima, Diederik van de Beek, Christina M.J.E. Vandenbroucke-Grauls, Wilbert Bitter, Jan-Willem Veening

**Affiliations:** 1 Amsterdam UMC Location Vrije Universiteit Amsterdam, Department of Medical Microbiology and Infection Prevention, De Boelelaan 1117, Amsterdam, the Netherlands; 2 Amsterdam Institute for Infection and Immunity, Amsterdam, the Netherlands; 3 Amsterdam UMC Location University of Amsterdam, Department of Neurology, Meibergdreef 9, Amsterdam, the Netherlands; 4 Amsterdam Neuroscience, Amsterdam, the Netherlands; 5 Department of Fundamental Microbiology, Faculty of Biology and Medicine, University of Lausanne, Biophore Building, 1015 Lausanne, Switzerland; 6 Section of Molecular Microbiology, Amsterdam Institute for Molecules, Medicines and Systems, VU University Amsterdam, 1081 Amsterdam, the Netherlands

**Keywords:** *Streptococcus pneumoniae*, *Danio rerio*, meningitis, host-pathogen interaction, dual RNA-seq, necroptosis, competence

## Abstract

Pneumolysin is a major virulence factor of *Streptococcus pneumoniae* that plays a key role in interaction with the host during invasive disease. How pneumolysin influences these dynamics between host and pathogen interaction during early phase of central nervous system infection in pneumococcal meningitis remains unclear. Using a whole-animal *in vivo* dual RNA sequencing (RNA-seq) approach, we identify pneumolysin-specific transcriptional responses in both *S*. *pneumoniae* and zebrafish (*Danio rerio*) during early pneumococcal meningitis. By functional enrichment analysis, we identify host pathways known to be activated by pneumolysin and discover the importance of necroptosis for host survival. Inhibition of this pathway using the drug GSK′872 increases host mortality during pneumococcal meningitis. On the pathogen’s side, we show that pneumolysin-dependent competence activation is crucial for intra-host replication and virulence. Altogether, this study provides new insights into pneumolysin-specific transcriptional responses and identifies key pathways involved in pneumococcal meningitis.

## Highlights

• Identification of pneumolysin-specific host-bacteria responses by whole-animal dual RNA-seq
• Identification of a functional necroptosis or necroptosis-like pathway in zebrafish
• Heterogeneity in competence development during infection
• Competence development is an important virulence determinant

## Graphical abstract

**Figure:**
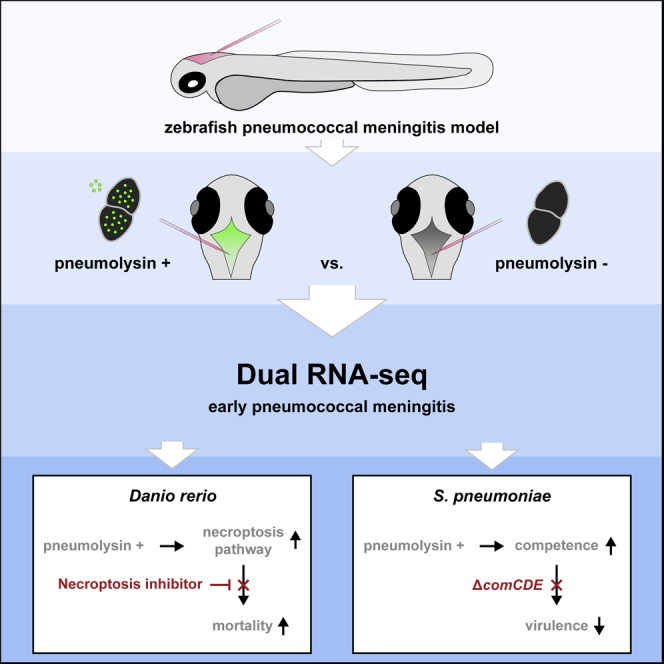

## Teaser

Jim et al. use a whole-animal *in vivo* dual RNA-seq approach to identify pneumolysin-specific transcriptional responses in both *Streptococcus pneumoniae* and zebrafish (*Danio rerio*) during early pneumococcal meningitis. Findings include the importance of necroptosis for host survival and the crucial role of pneumococcal competence activation for virulence.

## Introduction

The interaction between host and microbe determines the pathogenesis of infectious diseases. In invasive pneumococcal disease (IPD), caused by the opportunistic human pathogen *Streptococcus pneumoniae*, pneumococci need to successfully colonize mucosal surfaces of the nasopharynx by adapting to the host environment before they can spread into the blood stream, lungs, or central nervous system and cause IPD. Upon invasion, the pathogen will be detected by pattern recognition receptors (PRRs), leading to the activation of the innate immune response that will try to eliminate the pneumococci. In turn, pneumococci will counteract by employing a variety of strategies to escape from eradication by the host’s immune response.^1^^,^^2^^,^^3^^,^^4^ Insight into these complex host-pneumococcus interaction dynamics may lead to better understanding of the factors leading to poor disease outcome and to developing new intervention strategies.^5^ Despite the introduction of pneumococcal conjugate vaccines, the global burden of pneumococcal disease remains high, with an estimated 3.7 million episodes and up to half a million deaths every year in children under 5 years alone.^6^

To study infection dynamics, simultaneous profiling of both host and pathogen transcriptional responses, so-called dual RNA sequencing (RNA-seq), has been adopted as a tool that offers limited technical bias and higher efficiency compared with conventional approaches (single-species approach, array-based methods).^7^^,^^8^^,^^9^^,^^10^ In previous studies, using dual RNA-seq, we showed that *S*. *pneumoniae* represses the innate immune response in human alveolar lung epithelial cells and activates its own mucin-dependent sugar transporters upon adherence.^11^ This was also shown in a murine pneumonia model.^12^ So far, dual RNA-seq has mainly been applied in *in vitro* models of infection, which has led to valuable new insights into host-pathogen interactions for pathogens such as *Staphylococcus aureus*, *Plasmodium* spp., *Pseudomonas aeruginosa*, *Salmonella* spp., and *S*. *pneumoniae*.^9^^,^^11^^,^^13^^,^^14^^,^^15^^,^^16^^,^^17^^,^^18^^,^^19^ More recently, this approach has been also employed in *in vivo* infection models,^20^^,^^21^^,^^22^^,^^23^^,^^24^ although most of these experiments were often combined with some (flow-)sorting steps to identify infected cells. Recently, the *in vivo* pneumococcal and mouse transcriptomes within the nasopharynx, lungs, blood, heart, and kidneys were mapped, revealing distinct gene-expression profiles depending on organ and disease state.^25^ Together, these studies have demonstrated that dual RNA-seq can provide invaluable insights into host-microbe interactions.

Here, we applied whole-animal *in vivo* dual RNA-seq to specifically examine the role of pneumolysin on the transcriptional response of both pathogen (*S*. *pneumoniae* D39V) and host (*Danio rerio*) during the early phase of experimental pneumococcal meningitis, in which the initial innate immune response is activated, in a zebrafish infection model.^26^ Pneumolysin, a major pneumococcal virulence factor, is a cholesterol-dependent, pore-forming cytolysin produced by all known clinical isolates.^2^^,^^3^ It has been shown to play a role in the pathogenesis and pathophysiology of pneumococcal meningitis, and infection with pneumolysin-deficient mutants causes mild to no disease in experimental pneumococcal meningitis models.^26^^,^^27^^,^^28^^,^^29^^,^^30^ Pneumolysin might also play a key role in transmission by stimulating the host immune response.^2^ Moreover, persisting high levels of pneumolysin in the cerebrospinal fluid of patients with pneumococcal meningitis are associated with mortality.^31^ Pneumolysin was not required for pneumococcal replication in a murine influenza A virus-superinfection model of pneumonia,^32^ further highlighting our knowledge gap related to pneumolysin and its role in pathogenesis.

The serotype 2 *S*. *pneumoniae* strain D39 is one of the most used strains to study pneumococcal biology and infection. The genome of this strain, and a well-defined, genome-wide transcriptional response, have been annotated in detail.^33^^,^^34^ On the host side, the zebrafish has emerged as an *in vivo* model for various infections, including pneumococcal infections.^26^^,^^35^^,^^36^^,^^37^ Using whole-animal dual RNA-seq of a zebrafish model of *Shigella* infection, it was recently shown that *S*. *sonnei* virulence depends on its O-antigen oligosaccharide.^24^ Zebrafish have several advantages over mice, including small size and high fecundity; zebrafish embryos develop extra maternally and are optically clear, which makes them highly suitable for whole-organism-based high-throughput screening and real-time *in vivo* imaging.^36^^,^^37^ At a low dose of infection (300 colony-forming units [CFUs] of D39 pneumococci), zebrafish embryos develop disease progression and ultimately succumb to meningitis.^26^ In the present study, we identified known pathways activated by pneumolysin and novel pneumolysin-specific host-pathogen interactions in early pneumococcal meningitis, thereby providing a valuable resource for future studies. The data are easily accessible, searchable, and downloadable via a web-based platform (https://veeninglab.com/dual-danio). Moreover, we demonstrated that necroptosis is important for host survival in pneumococcal meningitis and that pneumococcal competence activation is a key hallmark of pneumococcal meningitis disease progression, delineating the essential role of pneumolysin in this process.

## Results

### Dual RNA-seq in early pneumococcal meningitis in zebrafish larvae

**Figure 1:**
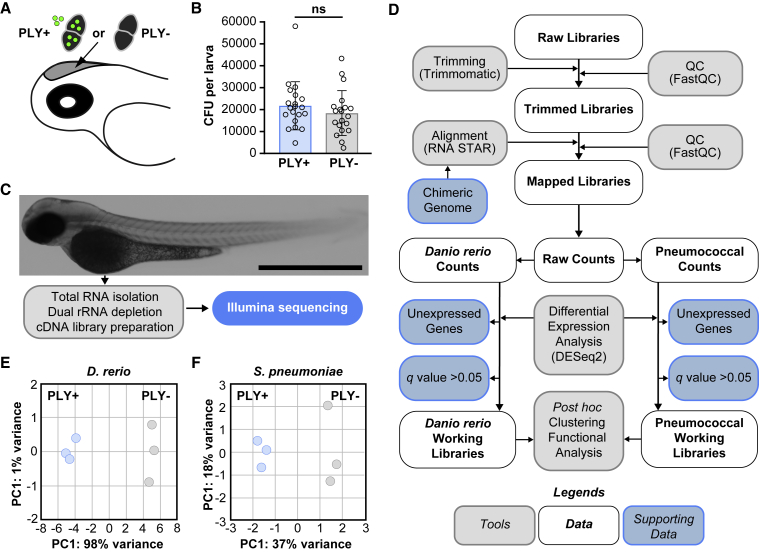
Dual RNA sequencing of host-pathogen transcriptomes in early pneumococcal meningitis (A) Zebrafish meningitis infection model. Pneumolysin-deficient *S*. *pneumoniae* D39V (PLY−) mutant bacteria or complemented *S*. *pneumoniae* D39V (PLY+) bacteria (∼2,000 CFUs) were injected into the hindbrain ventricle of 2 dpf zebrafish larvae. (B) Bacterial load in zebrafish larvae infected with *S*. *pneumoniae* PLY+ or *S*. *pneumoniae* PLY− at 8 hpi. The data represent the mean ± SD of two biological replicates with 9–10 larvae per group; each dot represents a single larva; ns, non-significant; determined by unpaired t test. (C) Total RNA was isolated from pooled infected zebrafish larvae (n = 100 per biological replicate) for preparation of cDNA libraries and sequencing at 8 hpi. Scale bar: 100 μm. (D) Quality control (QC) was performed on raw reads, low-quality reads were trimmed, and remaining reads were aligned to a synthetic chimeric genome. Aligned reads were counted and classified as pneumococcal or *D*. *rerio* counts. Final working libraries were created after removal of two gene fractions, and clustering and functional enrichment analysis were performed. (E) Principal-component (PC) analysis plot of pathogen transcriptional response to infection showed that the replicates cluster closely together. (F) PC analysis plot of host response showed similar clustering behavior.

To study host-pathogen interaction in early pneumococcal meningitis, we used our zebrafish embryo meningitis model and dual RNA-seq pipeline and performed RNA-seq simultaneously on both *D*. *rerio* and *S*. *pneumoniae*.^11^^,^^26^ To determine pneumolysin-specific transcriptional changes, we injected 2 days post fertilization (dpf) zebrafish larvae with a pneumolysin-deficient *S*. *pneumoniae* mutant strain (PLY−) or its complemented version (PLY+), in which the deleted pneumolysin gene is restored and thus able to produce pneumolysin (Figure 1A). Survival experiments showed that the complemented *S*. *pneumoniae* PLY+ strain regains its virulence compared with the pneumolysin-deficient mutant strain, albeit less virulence compared with the wild-type strain in the zebrafish embryo meningitis model, similar to what we have previously reported (Figure S1).^26^ Next, we wanted to determine the specific contributions of pneumolysin to transcription changes in the host and the pathogen. Considering the size of the host and the relatively small pathogen burden, the RNA signal will be dominated by *D*. *rerio* RNA. Therefore, we increased the inoculum of *S*. *pneumoniae* 3-fold (to 2,000 CFUs) compared with our previous work. Three biological replicates per group were used, with 100 larvae pooled per replicate (total n = 300 per group). Importantly, at 8 h post injection (hpi), the estimated total CFU of pneumococci per group per replicate was comparable between both groups (∼2 million CFUs *S*. *pneumoniae* per replicate; p = 0.3281) (Figure 1B). To minimize transcriptional changes caused by sample handling, we did not separate pneumococci from *D*. *rerio* cells before total RNA isolation and dual rRNA depletion (Figure 1C). The obtained dual RNA-seq dataset was pruned and handled as described previously (Figure 1D).^11^ In short, raw reads were trimmed and aligned to a chimeric concatenated genome of *D*. *rerio* and *S*. *pneumoniae*. After alignment, one-step mapping was performed to minimize false negatives. The resulting raw counts were counted separately and categorized as either *D*. *rerio* (ENSEMBL, release 11) or pneumococcal (D39V).^34^ Differential gene analysis was calculated by DESeq2, and specific groups of genes were removed from subsequent analysis.^38^ Finally, unbiased automated clustering and functional enrichment analysis were performed.^39^^,^^40^^,^^41^^,^^42^

**Figure 2:**
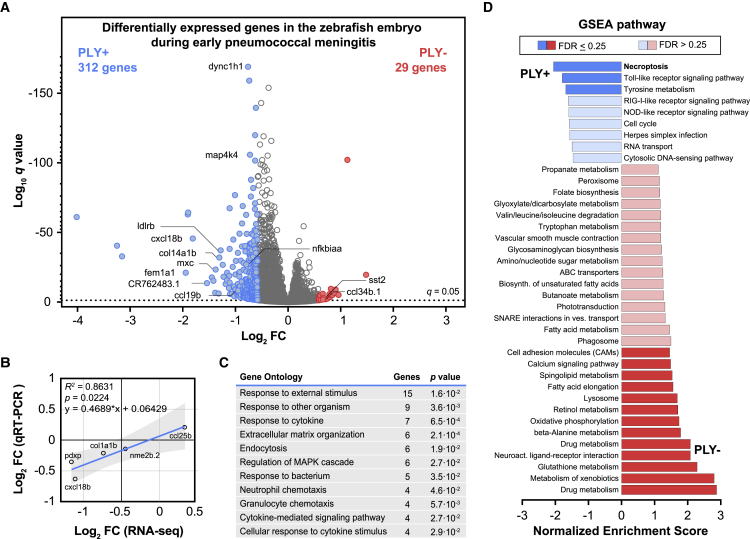
The host’s transcriptional response to infection with pneumolysin-positive or pneumolysin-negative *S*. *pneumoniae* D39V (A) Volcano plot from DESeq2 analysis of RNA pools from zebrafish larvae infected with the pneumolysin-deficient *S*. *pneumoniae* D39V mutant strain (PLY−) or the complemented *S*. *pneumoniae* D39V mutant strain (PLY+) at 8 hpi. The presence of pneumococcal pneumolysin activates a multitude of host genes in response to *S*. *pneumoniae* infection (FC > 1.5, *q* < 0.05). (B) Gene expression correlation between RNA sequencing (RNA-seq) and RT-qPCR data; each dot represents a single gene; correlation co-efficient (Pearson) and linear regression are indicated. (C) Gene Ontology enrichment analysis after DESeq2 analysis showed significant enrichment of several immunomodulatory pathways. Note that an individual gene can be part of multiple terms. (D) Gene set enrichment analysis after DESeq2 analysis showed enrichment of several immunomodulatory pathways in the presence of pneumolysin, whereas the phagolysosome pathway and many metabolic pathways were enriched in the absence of pneumolysin (FDR < 0.50).

Global descriptive analysis showed that the sizes of the six dual RNA-seq libraries were well-balanced (average ∼70 million reads, range: 55–99 million reads; Figure S2A). Moreover, 99.94% of reads could be aligned onto the zebrafish genome (99.94%–99.95%) with the remainder of the reads mapping to the pneumococcal genome. The number of reads translated into an average depth of 4.2 (3.3–5.9 times) for the host and 1.53 (1.22–2.35 times) for the pneumococcal libraries (Figure S2B). In addition, for all datasets, principal-component (PC) analyses showed that the replicates cluster close together (Figures 2E and 2F), indicating the homogeneity of response within the replicates and the transcriptional dissimilarity because of the presence or absence of pneumolysin. In the zebrafish dataset, there were 22,130 genes out of 25,592 (86%) in the raw library mapped (Figure S3; Table S1). After removal of unexpressed genes and genes with no significant difference (p < 0.05, *q* value by DESeq2), the zebrafish working libraries contained 6,403 genes (25%). In the pneumococcal libraries, out of 2,133 annotated genes, 1,924 genes (89%) were filtered and fed into downstream analysis (Figure S3; Table S2). The excluded 201 genes were not further processed because their mean normalized counts were less than the optimal threshold, as described by DESeq2.^38^

The raw data is publicly available at the Gene Expression Omnibus (GEO) under accession number GEO: GSE123988. To make the data more easily accessible, we also host the complete dual RNA-seq database online (https://veeninglab.com/dual-danio). On this website, users can select the gene of interest and download its expression level (Figure S4).

### Pneumolysin-specific host responses in pneumococcal meningitis

In the host transcriptional response, approximately 25% of the total zebrafish genes were differentially expressed at 8 hpi with *S*. *pneumoniae* PLY+ compared with zebrafish infected with *S*. *pneumoniae* PLY−. More specifically, 3,753 (14.7%) transcripts were significantly more abundant in zebrafish larvae infected with *S*. *pneumoniae* PLY+, whereas 2,650 genes (10.4%) were more abundant in zebrafish infected with *S*. *pneumoniae* PLY− (*q* < 0.05) (Figures 2A and S3). Gene expression values of five selected host transcripts were confirmed by RT-qPCR (Figure 2B). After applying a fold change (FC) cut-off at 1.5 for all comparisons, we identified 341 (1.3%) genes that are differentially expressed between both groups. The application of this more stringent cut-off resulted in a major shift of differentially expressed genes toward *S*. *pneumoniae* PLY+ infected larvae; in total, 312 genes (91.5%) were enriched in larvae infected with *S*. *pneumoniae* PLY+ compared with 29 (8.5%) genes in larvae infected with *S*. *pneumoniae* PLY− (Figure 2A; Table S1).

### Functional enrichment analyses identify several host pathways involved in pathogen detection and clearance

To analyze the differentially expressed genes, we performed functional enrichment analyses to determine their significance in the context of the background set of genes. First, we performed Gene Ontology (GO) biological process enrichment analysis (cut-off ≥ 3 genes, p < 0.05) on significantly enriched genes (*q* < 0.05, FC ≥ 1.5) in the presence (n = 312) or absence (n = 29) of pneumolysin to identify which GO terms were over-represented. In the presence of pneumolysin, mainly GO terms with genes known to be involved in immune signaling were identified, including “response to cytokine” (p = 6.3 × 10^−4^); “neutrophil chemotaxis” (p = 4.5 × 10^−3^); “granulocyte chemotaxis” (p = 5.6 × 10^−3^); “cytokine-mediated signaling pathways” (p = 2.6 × 10^−2^); and “cellular response to cytokine stimulus” (p = 2.9 × 10^−2^) (Figure 2C; Table S3). These genes encode for chemokines (Ccl19b, Cxcl18b), cytokine controlling transcription factors (June, Junba, Junbb), interleukin receptor (Il10rb), and other immunomodulatory proteins (Irak3, Mmp9, S1pr4, Tab1) (Table S1). Other enriched GO terms include “extracellular matrix structure” and “extracellular matrix organization,” which have been suggested to play a role in signaling and coordination of the migration of leukocytes, thus contributing to the inflammatory response against infections.^43^^,^^44^ In the absence of pneumolysin, we identified genes involved in terms associated with catabolic processes (Table S3).

Next, we performed gene set enrichment analysis (GSEA) at a low statistical cut-off (false discovery rate [FDR] < 0.50) to evaluate the differential gene expression data at the level of gene sets and to gain a rough insight in biologically relevant pathways and processes.^39^ GSEA also analyzes differentially expressed and functionally annotated genes in a dataset but discards the FC cut-off threshold limitation and uses a ranked list based on the direction and magnitude of expression change of these genes.^39^ Pathways known to be involved in intrinsic immune host defense mechanisms against pathogens were mainly enriched in zebrafish larvae infected with PLY+, including PRR pathways such as the Toll-like receptor (TLR) signaling pathway (FDR = 0.087), the RIG-I-like receptor (RLR) signaling pathway (FDR = 0.343), and the NOD-like receptor signaling pathway (FDR = 0.305) (Figure 2D; Table S3). At this stage of infection, the number of bacteria was comparable between both groups, suggesting that the enrichment of these pathways is due to the presence of pneumolysin (Figure 1B). PRRs are important receptors that recognize both exogenous as well as endogenous ligands, thereby contributing to the recognition of molecular structures of invading pathogens by the innate immune system.^45^ Moreover, these pathways are known to play a role in the defense against pneumococcal infection, and it has been shown that deficiency or knockout of gene(s) involved in these pathways often result in a more severe disease course during pneumococcal infection.^30^^,^^46^^,^^47^^,^^48^^,^^49^ Of note, in previous dual RNA-seq studies with *S*. *pneumoniae* using human lung epithelial cells or mouse models, the innate immune responses were also among the most upregulated gene-regulatory pathways.^11^^,^^25^ In the absence of pneumolysin, we identified many metabolic pathways, including fatty acid metabolism (FDR = 0.260), oxidative phosphorylation (FDR = 0.123), and glutathione metabolism (FDR = 0.004). Moreover, phagosome (FDR = 0.260) and lysosome (FDR = 0.131) pathways were enriched in larvae infected with PLY−, suggesting more active phagocytosis by the host in the absence of pneumolysin (Figure 2D; Table S3).

### Necroptosis pathway contributes to pathogen clearance

**Figure 3:**
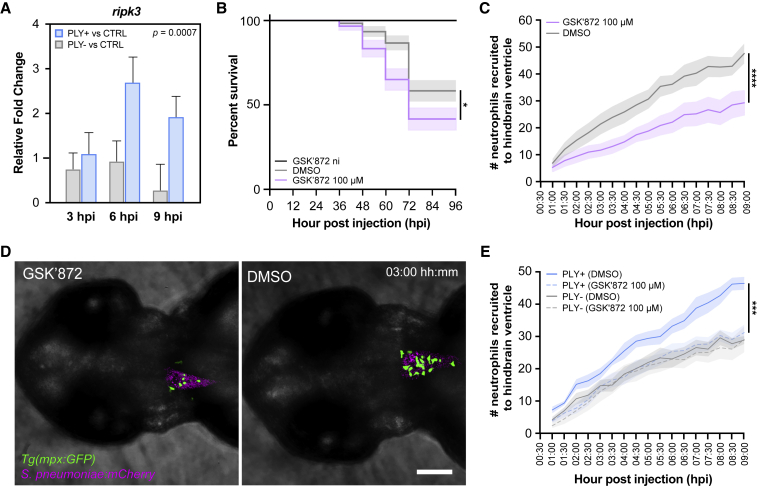
Inhibition of *ripk3* increases mortality in zebrafish larvae with pneumococcal meningitis and attenuates neutrophil recruitment during early stages of pneumococcal meningitis (A) Gene expression analysis of *ripk3* during early stages of pneumococcal meningitis. RT-qPCR was performed on pools of 20 zebrafish larvae injected with 2,000 CFUs of *S*. *pneumoniae* PLY+, *S*. *pneumoniae* PLY−, or PBS and collected at 3, 6, and 9 hpi. The data represent six biological replicates; F(1, 5) = 54.72, p = 0.0007, PLY+ versus control (CTRL) versus PLY− versus CTRL; determined by two-way ANOVA on log2-transformed values. Multiple comparison analyses of each individual time point showed no statistical differences between both groups: adjusted p = 0.9975, PLY+ versus CTRL versus PLY− versus CTRL at 3 hpi; adjusted p = 0.0676, at 6 hpi; adjusted p = 0.0829, at 9 hpi; determined by Sidak’s post hoc test on log2-transformed values. (B) Survival curves of 2 dpf zebrafish larvae injected with 200 CFUs of *S*. *pneumoniae* D39V into the hindbrain ventricle and control non-infected (ni) larvae treated with 100 μM GSK′872 or vehicle (DMSO). The data represent the mean ± SEM of three biological replicates with 20 larvae per group (n = 60 in total/group); ^∗^p = 0.0237; determined by log rank test. (C) Graph showing differences in number of neutrophils recruited to the hindbrain ventricle of zebrafish larvae injected with 2,000 CFUs of *S*. *pneumoniae* D39V upon treatment with 100 μM GSK′872 versus treatment with DMSO. The data represent the mean ± SEM of two biological replicates with 4–5 larvae per group (n = 9–10 in total/group); ^∗∗∗∗^p < 0.0001, GSK′872 (100 μM) versus vehicle (DMSO); determined by two-way ANOVA. (D) Representative confocal imaging of a single Z-slice of zebrafish larvae injected with 2,000 CFUs of red-fluorescently labeled *S*. *pneumoniae* into the hindbrain ventricle treated with 100 μM GSK′872 versus treatment with DMSO. Neutrophils are labeled by a GFP fusion to the *mpx* gene. Scale bar: 100 μm. (E) Graph of number of neutrophils recruited to the hindbrain ventricle of zebrafish larvae injected with 2,000 CFUs of *S*. *pneumoniae* PLY+ or *S*. *pneumoniae* PLY− and treatment with 100 μM GSK′872 versus treatment with DMSO. The data represent the mean ± SEM of two biological replicates with 5 larvae per group (n = 10 in total/group); ^∗∗∗^p = 0.0006, *S*. *pneumoniae* PLY+ (GSK′872, 100 μM) versus *S*. *pneumoniae* PLY+ (DMSO); p = 0.6441, *S*. *pneumoniae* PLY− (GSK′872, 100 μM) versus *S*. *pneumoniae* PLY− (DMSO); determined by two-way ANOVA.

A highly enriched immunoregulatory pathway in the presence of pneumolysin, which was less expressed in larvae challenged with the pneumolysin mutant, was the necroptosis pathway (FDR = 0.002) (Figure 2D; Table S3). Necroptosis is a caspase-independent form of programmed cell death that depends on activation of the key molecules receptor-interacting protein kinase 1 (RIPK1), RIPK3, and mixed-lineage kinase domain-like (MLKL) and has been shown to regulate inflammation.^50^^,^^51^^,^^52^ Both *ripk1l* (FC −1.09, *q* = 0.364) and *ripk3* (FC −1.08, *q* = 0.807) were enriched in the presence of pneumolysin-expressing bacteria, albeit not statistically significantly (Table S1). For MLKL, we could not identify a zebrafish ortholog (see discussion). To further validate that the putative necroptosis pathway is activated during pneumococcal infection, we performed RT-qPCR on *ripk3* expression over time, which is one of the key effectors of necroptosis. As shown in Figure 3A, *ripk3* was expressed to higher levels in larvae infected with *S*. *pneumoniae* PLY+ as compared to larvae infected with *S*. *pneumoniae* PLY− or phosphate-buffered saline (PBS) control-injected larvae as time progressed during the early stages of pneumococcal meningitis at 3, 6 and 9 hpi. To further study the role of this necroptosis or necroptosis-like pathway in pneumococcal meningitis, we infected zebrafish larvae with 200 CFUs of wild-type *S*. *pneumoniae* D39V in the hindbrain ventricle and treated them with the necroptosis pathway inhibitor GSK′872, which is an RIPK3 inhibitor.^53^^,^^54^ We decided to focus on RIPK3 because of its role as one of the key activators of necroptosis in the absence of MLKL. As shown in Figure 3B, inhibition of the necroptosis pathway by treatment with 100 μM GSK′872 resulted in a higher mortality rate in zebrafish larvae compared with vehicle (DMSO) treatment. Treatment of non-infected zebrafish with GSK′872 alone did not result in a higher mortality rate, suggesting that the inhibitor itself had no toxic effect at this concentration (Figure 3B).

The activation of necroptosis in neutrophils can lead to the release of damage-associated molecular patterns, resulting in an increased inflammatory innate response.^55^^,^^56^ One possibility for reduced zebrafish survival upon pneumococcal infection in the presence of the necroptosis inhibitor is perturbed signaling toward triggering the recruitment of dedicated immune cells, including neutrophils.^57^^,^^58^^,^^59^ To test this, we examined neutrophil recruitment to the infection site in the presence and the absence of GSK′872. As shown in Figure 3C, at 3 hpi (after injection with 2,000 CFUs of *S*. *pneumoniae* D39V), less neutrophils are visible in the hindbrain infection area. Quantitative time-lapse fluorescence microscopy corroborated this (Figure 3D). Pore-forming toxins can activate necroptosis in neutrophils as shown by Kitur et al.^60^ To investigate whether pneumolysin is involved in neutrophil necroptosis and recruitment, we infected zebrafish larvae with *S*. *pneumoniae* PLY+ or *S*. *pneumoniae* PLY−, followed by treatment with GSK′872 or DMSO, and performed quantitative time-lapse fluorescence imaging. We observed that zebrafish larvae infected with *S*. *pneumoniae* PLY− and treatment with GSK′872 did not result in a significant decrease in number of neutrophils recruited to the hindbrain ventricle compared with treatment with DMSO, whereas infection with *S*. *pneumoniae* PLY+ showed a similar trend to *S*. *pneumoniae* D39V wild-type infection (Figures 3C and 3E). These data suggest that pneumolysin not only plays a role in the recruitment of neutrophils to the site of infection but also in the activation of necroptosis in neutrophils. We hypothesize that in the absence of pneumolysin, necroptosis is less activated or not activated at all in neutrophils, hence there is no or a limited effect of GSK′872 on the recruitment of neutrophils to the hindbrain ventricle. Together, these data suggest that necroptosis plays a protective role in pneumococcal meningitis in our model, possibly by enabling correct signaling of dedicated immune cells to the site of infection (see below).

### Pneumolysin contributes to chemokine and cytokine signaling during infection

**Figure 4:**
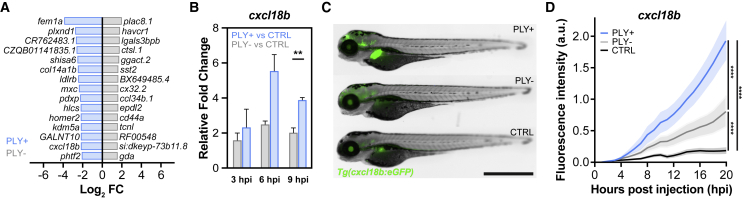
Cxcl18 expression in zebrafish larvae infected with pneumolysin-positive or pneumolysin-negative *S*. *pneumoniae* D39V (A) Top 20 highest significantly enriched genes in zebrafish infected with *S*. *pneumoniae* PLY+ or *S*. *pneumoniae* PLY− (*q* < 0.05). (B) Gene expression analysis of *cxcl18b* during early stages of pneumococcal meningitis. RT-qPCR was performed on pools of 20 zebrafish larvae injected with 2,000 CFUs of *S*. *pneumoniae* PLY+, *S*. *pneumoniae* PLY−, or control PBS injection and collected at 3, 6, and 9 hpi. The data represent four biological replicates; F(1, 6) = 44.21, ^∗∗^adjusted p = 0.0006, PLY+ versus CTRL versus PLY− versus CTRL; determined by two-way ANOVA on log2-transformed values; ^∗∗^p = 0.0063; determined by two-way ANOVA with Tukey’s post test on log2-transformed values. (C) Expression of Cxcl18b:EGFP is higher in zebrafish larvae (2 dpf) injected with 2,000 CFUs of *S*. *pneumoniae* PLY+ compared with infection with similar CFUs of *S*. *pneumoniae* PLY− or control PBS injection. Scale bar: 500 μm. (D) Quantification of Cxcl18b:EGFP expression in the head region over time. The data represent the mean ± SEM with 10 larvae per group; ^∗∗∗∗^p < 0.0001; determined by two-way ANOVA followed by Tukey’s post hoc test.

Among the most highly enriched transcripts in the presence of pneumolysin, we identified genes directly or indirectly involved in chemokine and cytokine signaling (e.g., *fem1a*, *plxnd1*, ENSDARG00000114454 [growth-regulated, alpha protein-like], *ldlrb*, *mxc*, and *cxcl18b*), confirming the role of pneumolysin as an important immunomodulatory virulence factor (Figures 2A and 4A).^3^^,^^61^^,^^62^^,^^63^^,^^64^^,^^65^ In the presence of pneumolysin, the most strongly enriched gene with known function was *fem1a* (FC −3.85, *q* = 7.55 × 10^−22^) (Figures 2A and 4A), also named EP4 receptor-associated protein. This protein is known to interact with nuclear factor κB (NF-κB) and suppresses inflammatory activation in human macrophages but also promotes inflammatory activation of murine microglia cells through mitogen-activated protein kinase 4-mediated signaling.^62^^,^^66^^,^^67^ Strikingly, in our dataset, NF-κB variants (*nfkbiaa*, *nfkbiab*, *nfkb1*, *nfkbz*) and *mapk4* were also significantly enriched in the presence of pneumolysin-expressing bacteria (Table S1). Other interesting genes involved in PRR signaling pathways that were enriched in zebrafish larvae infected with *S*. *pneumoniae* PLY+ compared with infection with *S*. *pneumoniae* PLY− are *irak4* (FC 1.80, *q* = 1.75 × 10^−9^) and *tbk1* (FC 1.41, *q* = 1.54 × 10^−18^) (Table S1). IRAK4 is the most extensively characterized TLR downstream signaling protein in invasive pneumococcal infection, and IRAK4 deficiency is associated with recurrent invasive pneumococcal infection in patients.^49^^,^^68^ TBK1 is an important downstream signaling molecule of the TLR, RIG-I/MDA-5, or cGAS-STING PRR pathway. In an experimental pneumonia mouse model, TBK1 knockout (KO) mice challenged intratracheally with *S. pneumoniae* showed a higher mortality rate compared with wild-type mice.^46^ Another enriched gene is *cxcl18b* (FC −2.16, *q* = 5.39 × 10^−39^) (Figures 2A and 4A; Table S1), a piscine- and amphibian-specific chemokine with similar function as Cxcl8a/interleukin-8 that exerts a neutrophil chemotactic function. However, these do not represent phagocytic cells but rather non-infected cells in tissue close to the site of infection.^65^ Interestingly, this gene, together with NF-κB variants, *il1β*, and other innate immunity signaling genes, were also found to be upregulated in a dual RNA-seq analysis of zebrafish larvae injected with *Shigella sonnei* into the hindbrain ventricle.^24^ To confirm that *cxcl18b* is indeed more highly expressed in the presence of pneumolysin-positive bacteria, we first performed gene expression analysis by RT-qPCR on whole zebrafish larvae at 3, 6, and 9 hpi. In zebrafish larvae injected with pneumococci, the expression of *cxcl18b* increased over time, with a significantly higher expression profile for zebrafish larvae injected with *S*. *pneumoniae* PLY+ in the hindbrain ventricle compared with an injection with a similar dose of *S*. *pneumoniae* PLY− or PBS control (Figure 4B). We then used the transgenic zebrafish line *Tg(cxcl18b*:*EGFP*), which allows for the visualization of *cxcl18b* expression, and observed that injection of *S*. *pneumoniae* PLY− bacteria induced *cxcl18b* expression above control levels (injection with just PBS) in the brain region over time (Figures 4C and 4D).^65^ However, injection with *S*. *pneumoniae* PLY+ resulted in a significantly higher expression of *cxcl18b* compared with injection with *S*. *pneumoniae* PLY− or injection with PBS, in line with the RT-qPCR data (Figures 4C and 4D; Video S1).

Video S1. Differences in Cxcl18b:EGFP expression over time in zebrafish larvae injected with *S*. *pneumoniae* versus control injection with PBS Zebrafish larvae were injected with 2,000 CFU of *S*. *pneumoniae* PLY+ or PBS into the hindbrain ventricle at 2 days post fertilization. Interval time between frames is 1 h for 20 h. Frame rate is 2 frames per s. Scale bar: 100 μm.

### Pneumolysin-specific pneumococcal transcriptional changes in early pneumococcal meningitis

**Figure 5:**
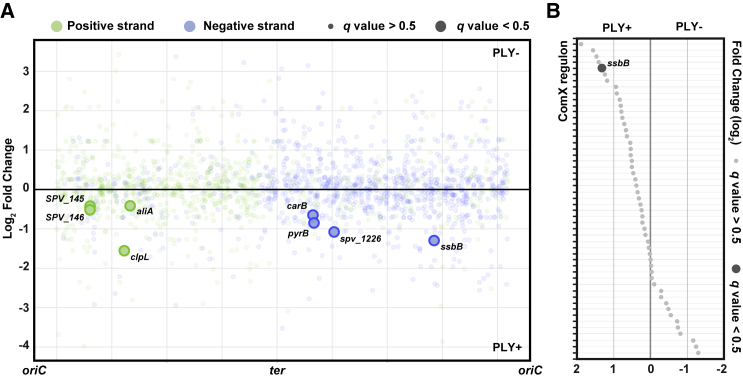
Transcriptional response in pneumolysin-positive or pneumolysin-negative *S*. *pneumoniae* D39V (A) Pneumolysin-specific transcriptional rewiring in *S*. *pneumoniae* in response to injection in zebrafish embryo. Fold change of transcriptional response in *S*. *pneumoniae* PLY+ and PLY− is plotted against its genomic location. (B) Fold change of ComX regulons between *S*. *pneumoniae* PLY+ and *S*. *pneumoniae* PLY− strains shows gene expression regulation in response to zebrafish infection. *ssbB*, a member of the regulon shows increased expression in *S*. *pneumoniae* PLY+ compared with *S*. *pneumoniae* PLY− (FC ≥ 2.0, *q* < 0.5). Regulons are sorted by fold change value in descending order.

Due to the relative lower number of reads of the pneumococcal transcriptome compared with the zebrafish transcriptome, we applied a softer cut-off (limiting only the p value, *q* < 0.5 without FC limitation) to obtain a more complete impression of the role of pneumolysin during early pneumococcal meningitis (Table S2). Using this cut-off, eight pneumococcal genes were differentially expressed between *S*. *pneumoniae* PLY+ and *S*. *pneumoniae* PLY− (*q* < 0.5); all of them were expressed more in the *S*. *pneumoniae* PLY+ strain (Figure 5A). The eight genes code for the Clp protease component ClpL; transporters for oligopeptides (AliA) and amino acids (GlnH); enzymes in pyrimidine biosynthesis cascade (PyrB-CarB); competence-induced single-stranded DNA binding protein (SsbB); and two hypothetical proteins (SPV_0145-6), one of which is putatively encoding a metalloproteinase. Since *aliA* is under the regulation of CodY, a global regulator of protein metabolism, and *glnH* is under the regulation of ArgR, an arginine repressor, the *S*. *pneumoniae* PLY+ strain possibly encounters different nutrients than the *S*. *pneumoniae* PLY− strain. This hypothesis is further supported by the higher expression of *pyrB-carB*; both genes code for enzymes in the pyrimidine biosynthesis cascade and are organized in one operon under PyrR regulation, which in turn is sensitive to the levels of intracellular uridine (Figure 5A).^34^

The higher expression of *ssbB*, a ComX regulon, indicates that at least a subset of *S*. *pneumoniae* PLY+ bacteria is inducing competence genes inside the host.^69^ Competence activation is consistent with the expression of *clpL*, as previously observed.^33^^,^^69^ In addition to *ssbB*, 37 other competence genes were expressed at higher levels in the *S*. *pneumoniae* PLY+ strain, albeit not statistically significant (Figure 5B). Surprisingly, a small subset of competence genes (16) was also upregulated in pneumolysin-deficient *S*. *pneumoniae* (Figure 5B).

### Competence development promotes virulence during early meningitis

**Figure 6:**
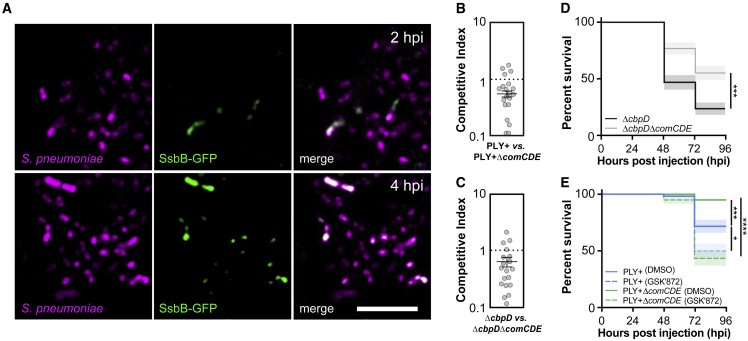
Pneumococcal competence in zebrafish larvae with pneumococcal meningitis (A) Single-plane confocal microscopy images showing increased heterogeneous expression of SsbB-GFP over time in constitutively HlpA-mCherry-expressing pneumococci injected into the hindbrain ventricle of 2 dpf zebrafish larvae. Scale bar: 10 μm. (B) Competitive index (CI) analysis of 2 dpf larvae co-injected with similar number of *S*. *pneumoniae* D39V PLY+ (HlpA-GFP) and *S*. *pneumoniae* D39V PLY+ Δ*comCDE* (HlpA-mKate2) (B) and CI of larvae co-injected with similar number of *S*. *pneumoniae* D39V Δ*cbpD* and *S*. *pneumoniae* D39V Δ*cbpD*Δ*comCDE.* (C) Larvae were harvested at 24 hpi. A CI score of 1 denotes no difference in virulence. The data represent the mean ± SEM of two biological replicates with 10 larvae per group; each dot represents a single larva; p < 0.0001, D39V PLY+ versus D39V PLY+ Δ*comCDE*; p < 0.01, D39V Δ*cbpD* versus Δ*cbpD*Δ*comCDE*; determined by one sample t test. (D) Survival curves of 2 dpf zebrafish larvae injected with 400 CFUs of *S*. *pneumoniae* D39V PLY+ or *S*. *pneumoniae* D39V PLY+ Δ*comCDE* into the hindbrain ventricle. The data represent the mean ± SEM of three biological replicates with 20 larvae per group (n = 60 in total/group); ^∗∗∗^p = 0.0001; determined by log rank test. (E) Survival curves of 2 dpf zebrafish injected with 300 CFUs of *S*. *pneumoniae* D39V PLY+ or *S*. *pneumoniae* D39V PLY+ Δ*comCDE* into the hindbrain ventricle and treatment with 100 μM GSK′872 (RIPK3 inhibitor) or vehicle (DMSO). The data represent the mean ± SEM of three biological replicates with 20 larvae per group (n = 60 in total/group); ^∗^p = 0.0177, ^∗∗∗^p = 0.0006, ^∗∗∗∗^p < 0.0001; determined by log rank test.

The *ssbB* gene is under direct control of the competence sigma factor ComX, strongly correlates with the incorporation of external DNA, and is a good reporter for competence.^70^^,^^71^ To monitor whether *ssbB* is activated during early pneumococcal meningitis, we constructed a reporter strain that expresses GFP upon *ssbB* activation (SsbB-GFP). As a control, this strain also constitutively expresses RFP. Next, the competence-reporter strain was injected into the hindbrain ventricle of zebrafish larvae. From as early as 2 hpi onward, we observed heterogeneous SsbB-GFP expression, which increases over time in the bacterial population growing at the site of injection, demonstrating that competence is activated in part of the bacterial population during early pneumococcal meningitis (Figure 6A).

We hypothesize that competence plays a role in virulence in pneumococcal meningitis. To study whether competence influences virulence in pneumococcal meningitis, we constructed a *comCDE* KO mutant strain in the *S*. *pneumoniae* PLY+ background, as this operon controls competence regulation.^72^ First, we performed a competitive index experiment by co-injecting both the complemented PLY+ and PLY+Δ*comCDE* strains into the zebrafish embryo hindbrain with a similar number of bacteria and determined the number of bacteria before injection and at 24 hpi. We observed that the number of CFU of *S*. *pneumoniae* PLY+Δ*comCDE* (HlpA-mKate2) present in zebrafish larvae at 24 hpi was lower compared with *S*. *pneumoniae* PLY+ (HlpA-GFP), suggesting that deletion of the *comCDE* operon resulted in decreased fitness compared with the *S*. *pneumoniae* PLY+ strain (p < 0.0001) (Figure 6B). To exclude the possibility that this decreased fitness is caused by fratricins produced by competent wild type-bacteria causing lysis of the Δ*comCDE* mutant,^73^^,^^74^ we constructed an *S*. *pneumoniae* D39V Δ*cbpD* mutant that is unable to produce the major fratricin, i.e., murein hydrolase CbpD. We compared this mutant in a competition experiment with an *S*. *pneumoniae* D39V Δ*cbpD*Δ*comCDE* double mutant. Consistent with the previous experiment, the number of CFUs of *S*. *pneumoniae* D39V Δ*cbpD*Δ*comCDE* was also lower compared wtih *S*. *pneumoniae* D39V Δ*cbpD* (p < 0.01) (Figure 6C), in line with the observation that competence is induced in spatially clustered foci within the zebrafish (Figure 6A). Additionally, we also performed individual survival experiments with the separate strains. After injection with *S*. *pneumoniae* D39V Δ*cbpD*Δ*comCDE*, we observed a significantly lower larvae mortality rate compared with injection with *S*. *pneumoniae* D39V Δ*cbpD* (p = 0.0001) (Figure 6D). These results are in line with recent findings in a murine model of pneumococcal meningitis.^75^ To investigate whether competence also plays a role in necroptosis, we infected larvae with *S*. *pneumoniae* D39V PLY+ or *S*. *pneumoniae* D39V PLY+Δ*comCDE* followed by treatment with 100 μM GSK′872 or vehicle (DMSO) (Figure 6E). Strikingly, we observed that upon treatment with GSK′872, the survival rate of zebrafish larvae infected with the isogenic *S*. *pneumoniae* D39V PLY+ Δ*comCDE* is reduced substantially, similar to zebrafish larvae infected the *S*. *pneumoniae* D39V PLY+ treated with GSK′872 (p < 0.0001; determined by log rank test). This suggests that neutrophils play an important role at this stage of the infection. Recent work from our group has shown that competence development is important for the display of pneumococcal surface proteins.^76^ These surface proteins (i.e., PspA and PspC) play an important role in the defense against the innate immune system by inhibiting opsonization and complement evasion, which hampers the efficient recognition by neutrophils.^77^^,^^78^ The data shown here suggest that in the case of a normal influx of neutrophils, the competence mutant is not able to establish a lethal infection in most larvae and that pneumococci are rapidly cleared. However, in the presence of GSK′872, which results in reduced neutrophil influx, even the competence mutant can build up critical mass that then can go on to cause a lethal infection. Together, these results indicate that the *comCDE* operon, and therefore competence, is involved in pneumococcal virulence.

## Discussion

We present a detailed dataset of pneumolysin-specific zebrafish-pneumococci transcriptional responses in early pneumococcal meningitis using whole-animal *in vivo* dual RNA-seq. This whole-animal approach alleviates the need for dissecting and/or sorting of infected tissues, thereby reducing experimental handling and technical noise. Our experimental setup allowed us to identify not only previously reported but also novel pneumolysin-specific transcriptional responses in both host and pathogen. We found that in the presence of pneumolysin, major immunomodulatory pathways were enriched and that competence plays a role in pneumococcal virulence in pneumococcal meningitis among others.

Activation of the immune system in the cerebrospinal fluid is dependent on PRRs.^30^^,^^79^ In our study, we found that genes involved in innate immune signaling and PRR pathways like the TLR signaling pathway, the RLR pathway, and the NOD-like receptor signaling pathway were enriched in zebrafish larvae injected with pneumococci that produce pneumolysin in the hindbrain ventricle (Figure 2D; Table S2). Malley et al. have shown that in mice, the TLR4 signaling pathway is responsible for recognition of pneumococcal pneumolysin.^80^ In zebrafish, however, there is evidence that TLR4 proteins do not function in a similar fashion as in mammals.^81^^,^^82^ The only *tlr4*-like gene we found significantly enriched was *tlr4ba* (FC 1.31, *q* = 0.038) (Table S1) and only in zebrafish infected with the pneumolysin-deficient mutant strain, suggesting that the recognition of pneumolysin in early pneumococcal meningitis in zebrafish most likely depends on a different PRR pathway. Previous studies have shown that the NLRP3 inflammasome is one of the PRR pathways that can be activated by pneumolysin, which results in the secretion of IL-1β via caspase 1.^48^ In our dataset, we found that *il1β* (FC −1.22, *q* = 0.017) (Table S1) was significantly more highly expressed in zebrafish larvae infected with pneumolysin-positive pneumococci compared with those infected with pneumolysin-deficient pneumococci. However, both zebrafish orthologs for NLRP3 (*nlrp3*; FC 1.05, *q* = 0.707) (Table S1) and caspase 1 (*caspa*; FC −1.06, *q* = 0.636) (Table S1) were statistically not significantly differentially expressed between both groups, while ASC/PYCARD (*pycard*; FC 1.10, *q* = 0.017) was downregulated in the presence of pneumolysin. This suggests that the increased expression of *il1β* in zebrafish infected with pneumolysin-positive pneumococci is possibly NLRP3 independent and due to another PRR pathway, such as the aforementioned RIG-I-like pathway or the cGAS/STING pathway with TBK1 as the central signaling molecule.^46^^,^^83^

Necroptosis is a caspase-independent programmed form of necrosis that regulates inflammation and has been implicated to play an important role in infectious disease pathology.^50^^,^^51^^,^^52^ Interestingly, the core necroptotic machinery in mammals consists of RIPK3 and MLKL, the latter of which is not present in zebrafish.^84^ Nevertheless, inhibition of the necroptosis pathway with GSK′872 resulted in a higher mortality rate in infected zebrafish larvae (Figure 3B). This corroborates the importance of an intact PRR pathway in the host defense against pathogens as shown previously by other studies.^30^^,^^46^^,^^47^^,^^48^^,^^49^ Furthermore, this suggests that zebrafish have an alternative cell death executor instead of MLKL or that RIPK3 triggers apoptosis. Alternatively, RIPK1-RIPK3 activation may restrict bacterial proliferation by a different pathway, for instance by triggering inflammation independent of MLKL.^85^ Recent studies show that diverse bacterial pore-forming toxins, including pneumolysin, can also induce necroptosis in neutrophils, macrophages, and lung epithelial cells.^86^^,^^87^^,^^88^ Notably, we found that the necroptosis pathway was one of the most enriched pathways in zebrafish larvae injected with the pneumolysin-positive pneumococci, indicating that pneumolysin plays a role in activating necroptosis in pneumococcal meningitis, most likely via neutrophils (Figures 3C–3E). The role of necroptosis in infection remains a topic of debate. The study of Kitur et al. showed that necroptosis limits pathological inflammation and enhances survival in a murine *S*. *aureus* skin and sepsis model,^89^ whereas other studies have shown that pore-forming, toxin-induced necroptosis exacerbates pulmonary injury and is detrimental to the host during bacterial pneumonia.^60^^,^^87^^,^^88^ Elucidating the role of necroptosis may provide new avenues for treatment of infectious diseases.

Finally, we provide the first visualization of pneumococcal competence development *in vivo* at the single-cell level. This shows that, in contrast to the *in vitro* situation where all cells within a clonal population synchronously become competent, during meningitis in zebrafish, not all pneumococcal cells develop competence at the same time (Figure 6A).^71^^,^^90^^,^^91^^,^^92^ It also challenges the idea that competence is constitutively expressed by individual bacteria in the host; instead, there are pockets of pneumococci inducing competence during infection (Figure 6A).^93^ This is in line with evidence that cell-to-cell communication and cell-to-cell heterogeneity are of major importance in the pathogenesis of infectious diseases.^94^^,^^95^ Competence in *S*. *pneumoniae* is regulated by a quorum-sensing system and is triggered by chemical signals and environmental factors such as antibiotic-induced replication stress, environmental cues, cell density, and cell history.^71^^,^^92^ This system operates via the secreted peptide competence stimulating factor (CSP). Inactivation of the *comCDE* operon, which encodes the CSP precursor, histidine kinase, and a response regulator results in complete abolishment of competence development.^71^ While competence allows for DNA uptake, most of the genes that are part of the competence regulon are not involved in transformation and likely function as a general stress response.^96^^,^^97^ We observed that deletion of *comCDE* attenuated virulence in pneumococcal meningitis, which is in line with recent work that also showed a key role for competence in a murine meningitis model (Figures 6B–6E).^75^ Moreover, in our previous dual RNA-seq study we also observed that competence plays an important role during early infection of epithelial cells *in vitro*. 11 Interestingly, a recent study showed that to successfully infect the host, *S*. *pneumoniae* heterogeneously expresses pneumolysin to overcome host defense strategies and cross the blood-brain barrier by rising different bacterial subpopulations that can either attack or evade autophagosomes.^29^ Moreover, both competence as well as heterogeneity have been reported to play a role in streptococcal biofilm formation, an important virulence factor for nasopharyngeal colonization, pneumonia, and otitis media.^98^^,^^99^^,^^100^ This suggests that cell-to-cell communication and cell-to-cell heterogeneity in pneumococci are indeed involved in the pathogenesis of pneumococcal infection. It remains to be elucidated how the heterogeneous competence expression is involved, whether directly or indirectly, in pneumolysin-related virulence.

In summary, our work provides a detailed view of host-pathogen transcripts during early pneumococcal meningitis. The transcriptome data can serve as a valuable resource for future studies, with an emphasis on genes and pathways that we did not elaborate on in this study. Moreover, we made the dataset available online on https://veeninglab.com/dual-danio and encourage other researchers to use the dataset to further validate research findings and develop new hypotheses. Understanding the role and mechanism of pneumolysin in host-pathogen interactions will provide clues for future treatment strategies to combat not only pneumococcal meningitis but also other forms of IPD.

### Limitations of the study

Our study has several limitations. To begin, because of the low read depth of the pneumococcal transcriptomes, the number of statistically significantl*y* differentially expressed pneumococcal genes was relatively low compared with the zebrafish transcriptomes. Future studies may benefit from increasing the number of larvae by increasing the infectious inoculum and/or the use of more sensitive low-input RNA-seq methods to improve the read depth of pneumococci, thereby potentially identifying more biologically relevant pneumococcal genes. Furthermore, although GSK′872 appears to be a specific RIPK3 inhibitor with minimal cross-reactivity, we cannot rule out downstream effects on other cell death pathways in our study. Moreover, although our findings suggest that necroptosis or the necroptosis-like pathway plays an important role in the host defense against pneumococci during pneumococcal meningitis, we could not identify a zebrafish ortholog for MLKL. Hence, our results should be interpreted with caution as it may only be partially translatable to humans.

## STAR★Methods

### Key resources table

**Table:** 

REAGENT or RESOURCE	SOURCE	IDENTIFIER
**Bacterial and virus strains**		

*Streptococcus pneumoniae* D39V: serotype 2	Avery et al.^101^ Slager et al.^34^	N/A
*Streptococcus pneumoniae* D39V *hlpA_hlpA*-mCherry cam^r^	Beilharz et al.^102^	MK218
*Streptococcus pneumoniae* D39V Δ*ply*::camr, CIL::*ply*	This paper	LK01
*Streptococcus pneumoniae* D39V Δ*ply*::camr, CIL::kan^r^	This paper	RA49
*Streptococcus pneumoniae* D39V *ssbB*-GFP, *hlpA_hlpA*-mCherry_cam^r^	This paper	KJ43
*Streptococcus pneumoniae* D39V Δ*ply*::camr, CIL::*ply*, hlpA_hlpA-mKate2::trmp^r^	This paper	VL2725
*Streptococcus pneumoniae* D39V Δ*ply*::cam^r^, CIL::*ply*, Δ*comCDE*::ery^r^, *hlpA*-GFP::trmp^r^	This paper	VL2728
*Streptococcus pneumoniae* D39V Δ*cbpD*::spec^r^, *ΔcomCDE*::cam^r^	This paper	ADP352
*Streptococcus pneumoniae* D39V Δ*cbpD*::spec^r^	This paper	VL561

**Chemicals, peptides, and recombinant proteins**		

1-phenyl 2-thiourea (PTU)	Sigma-Aldrich	Cat# P7629
CSP-1	AnaSpec	Cat# AS-63779
DNase I recombinant, RNase-free	Roche	Cat# 4716728001
GSK′872	MedChemExpress	Cat# HY-101872
Streptococcus selective supplement (COBA)	Oxoid	Cat# SR0126
Tricaine	Sigma-Aldrich	Cat# A5040

**Critical commercial assays**		

NucleoSpin RNA isolation kit	Machery-Nagel	Cat# 740955.50
SuperScript® IV VILO	Invitrogen	Cat# 11756050

**Deposited data**		

Dual RNA-seq dataset	This paper	GSE123988 (https://www.ncbi.nlm.nih.gov/geo/query/acc.cgi?acc=GSE123988)
*Streptococcus pneumoniae* D39V genome	Genome Reference Consortium	GCF_003003495 (https://www.ncbi.nlm.nih.gov/assembly/GCF_003003495.1/)
*Danio rerio* (zebrafish) genome NCBI build 11, GRCz11	Genome Reference Consortium	http://ftp.ensembl.org/pub/release-94/fasta/danio_rerio/dna/

**Experimental models: Organisms/strains**		

Zebrafish: *casper*	D’agati et al.^103^ White et al.^104^	ZFIN: ZDB-FISH-150901-6638
Zebrafish: *Tg(cxcl18b*:*EGFP)*	Torraca et al.^65^	ZFIN: ZDB-FISH-170410-1
Zebrafish: *Tg(mpx*:*GFP)*	Renshaw et al.^105^	ZFIN: ZDB-FISH-161202-8

**Oligonucleotides**		

Primers for construction of bacterial mutant strains, see Table S4	This paper	N/A
Primers for RT-qPCR, see Table S4	This paper	N/A

**Software and algorithms**		

FastQC v0.11.8	Babraham Bioinformatics	https://www.bioinformatics.babraham.ac.uk/
featureCounts v1.6.3	Liao et al.^106^	https://subread.sourceforge.net/
Illustrator	Adobe	https://www.adobe.com/
ImageJ	Schneider et al.^107^	https://imagej.nih.gov/ij
InDesign	Adobe	https://www.adobe.com/
Prism 8.0	GraphPad	https://www.graphpad.com/
R v3.5.2	The R Project for Statistical Computing	https://www.r-project.org/
RNA-STAR v.2.6.0a	Dobin et al.^108^	https://bioinformaticshome.com/
Trimmomatic v0.38	Bolger et al.^109^	http://www.usadellab.org/
WebGestalt 2019	Liao et al.^42^	http://www.webgestalt.org/

**Other**		

Dual RNA-seq via a web-based platform	This paper	https://veeninglab.com/dual-danio

### Resource availability

#### Lead contact

Further information and requests for resources and reagents should be directed to and will be fulfilled by the lead contact, Jan-Willem Veening (Jan-Willem.Veening@unil.ch).

#### Materials availability

This study did not generate new unique reagents. Information on reagents used in this study is available in the key resources table.

### Experimental models and subject details

#### Bacterial strains and growth conditions

All pneumococcal strains used in this study are derivatives of the clinical isolate *S*. *pneumoniae* D39 from the Veening lab (D39V), unless specified otherwise and are listed in the Key resources table.^34^^,^^101^ Oligonucleotides are listed in Table S4. *S*. *pneumoniae* was grown at 37°C on Columbia agar blood plates supplemented with 5% sheep blood (bioMérieux; Cat# 43049) or in C + Y medium.^110^ For transformation, pneumococcal cells were grown in C + Y medium at 37°C to an OD595 of approximately 0.100. Subsequently, 100 ng/mL of synthetic CSP-1 (AnaSpec, Cat# AS-63779) was added and the cells were incubated for 10 min at 37°C. DNA was added to the activated cells and incubated for 20 min at 30°C. Cells were then diluted 10 times in fresh C + Y medium and incubated for 1.5 h at 37°C. Transformants were selected by plating on Columbia agar blood plates supplemented with 2% (v/v) defibrinated sheep blood with the appropriate antibiotics. Antibiotic concentrations for selection used for *S*. *pneumoniae* were: erythromycin (ery) 0.25 μg/mL, chloramphenicol (cam) 4.5 μg/mL, kanamycin (kan) 250 μg/mL, spectinomycin (spec) 100 μg/mL, and trimethoprim (trmp) 20 μg/mL.

#### Construction of pneumolysin deficient S. pneumoniae D39V PLY- mutant strain and its complemented S. pneumoniae D39V PLY+ strain

To construct the pneumolysin-deficient mutant strain PLY-, we transferred the pPEPY plasmid, which integrates at the chromosomal integration locus (CIL) in a non-coding region of the pneumococcal genome, to a previously published pneumolysin deficient mutant strain,^111^^,^^112^ resulting in strain RA49. The isogenic complemented strain PLY- was constructed by ectopic integration of the PEPY plasmid with pneumolysin under native promoter and a kanamycin marker, resulting in strain LK01. This construct was made by amplifying the pneumolysin promoter from genomic *S*. *pneumoniae* D39V DNA with primer set LK283 and LK284. Pneumolysin with native terminator was amplified from genomic D39 DNA using primer set LK285 and LK286. Terminal primers LK283 and LK286 were used to join the two fragments together and contain restriction sites BamHI and XbaI respectively.

#### Construction of fluorescently labeled S. pneumoniae D39V ΔcomCDE mutant strain and its complemented strain

To construct the *comCDE* deficient mutant strain, erythromycin resistance marker (*ery*^*r*^) was amplified with using primers OVL2549 and OVL2771 from genomic DNA of the *hexA*::*ery*^*r*^ strain (Veening lab collection). The upstream region of *comCDE* was amplified using primers OVL506 and OVL2548, the downstream region with primers OVL2773 and OVL1667 using genomic DNA of *S*. *pneumoniae* D39V as template. The resulting three fragments were fused by Golden Gate cloning with BsmBI, and transformed into the complemented *S*. *pneumoniae* D39V PLY+ strain. Transformants were selected on Columbia blood agar containing 0.25 μg/mL of erythromycin, and correct colonies were verified by PCR and sequencing. To fluorescently label *S*. *pneumoniae* strains that already have a chloramphenicol resistance marker (cam^r^), we replaced cam^r^ with trimethoprim resistance marker (trmp^r^) on *hlpA-sfGFP/RFP_cam*^*r*^ constructs. The *trmp*^*r*^ gene was amplified using primers OVL2549 and OVL2772 from genomic DNA of the *hexA*::*trmp*^*r*^ strain (Veening lab collection). Using genomic DNA of JWV500 (*hlpA*::*hlpA-sfGFP_cam*^*r*^)^113^ or MK119 (*hlpA*::*hlpA_hlpA-mKate2_cam*^*r*^),^114^ the upstream fragment containing *hlpA-sfGFP or hlpA_hlpA-mKate2* was amplified by PCR with primers OVL43 and OVL2769, and the downstream fragment of *cam*^*r*^ gene containing transcriptional terminator was amplified using primers OVL2770 and OVL46. The resulting three fragments were fused by Golden Gate cloning with *BsmB*I, and transformed into the respective *S*. *pneumoniae* strains, resulting in strains VL2725 and VL2728. Transformants were selected on Columbia blood agar containing 20 μg/mL of trimethoprim, and correct colonies were verified by PCR and sequencing.

#### Construction of SsbB-GFP, HlpA-mCherry reporter strain

To monitor competence development of pneumococci *in vivo*, we transformed the amplified PCR product of the *hlpA_hlpA*-mCherry fragment and a downstream chloramphenicol cassette using chromosomal DNA of strain MK218 as a template into a strain containing a translational fusion of GFP to the competence-induced SsbB protein.^11^^,^^102^ This strain, KJ43, expresses constitutively mCherry and expresses SsbB-GFP upon competence induction. Transformants were selected on Columbia blood agar containing 4.5 μg/mL of chloramphenicol.

#### Construction of ΔcbpD and isogenic ΔcbpDΔcomCDE mutant strains

To construct the Δ*cbpD* mutant strain, the gene *cbpD*, encoding the fratricin called choline-binding protein D, was replaced by a spectinomycin resistance (spec^r^) marker. The upstream region was amplified using primers ADP1/45 and ADP1/46 + SphI, the downstream region with primers ADP1/47 + HindIII and ADP1/48, and the spectinomycin resistance marker with sPG11 + SphI and sPG12 + HindIII. All three fragments were digested with the proper restriction enzymes (SphI and/or HindIII) and ligated together. The Δ*cbpD*:spec fragment containing the spectinomycin resistance marker flanked by the sequence up- and downstream of *cbpD* was transformed into *S*. *pneumoniae* D39V resulting in strain VL561. Transformants were selected on Columbia blood agar containing 200 μg/mL spectinomycin. Correct deletion was verified by PCR and sequencing. To construct the isogenic Δ*cbpD*Δ*comCDE* mutant strain, the Δ*comCDE*:cat fragment from strain ADP107 was transformed into VL561 resulting in strain ADP352.^92^ All transformants were selected on Columbia blood agar containing 3.5 μg/mL of chloramphenicol, and correct colonies were verified by PCR and sequencing.

#### Zebrafish husbandry and maintenance

Transparent adult *casper* mutant zebrafish, *Tg(mpx*:*GFP) casper* zebrafish expressing green fluorescently labeled neutrophils, and *Tg(cxcl18b*:*eGFP)* wild-type zebrafish expressing green fluorescence upon induction of the inflammatory marker chemokine cxcl18b were maintained at 26°C in aerated 5-L tanks with a 10/14 h dark/light cycle.^65^^,^^103^^,^^104^^,^^105^ Zebrafish embryos or larvae were raised at 28°C in a temperature-controlled incubator in E3 medium (5.0 mM NaCl, 0.17 mM KCl, 0.33 mM CaCl_2_·2H_2_O, 0.33 mM MgCl_2_·6H_2_O) supplemented with 0.3 mg/L methylene blue. If necessary, *Tg(cxcl18b*:*eGFP)* wild-type zebrafish were additionally treated with 0.003% (v/v) 1-phenyl 2-thiourea (PTU) (Sigma-Aldrich; Cat# P7629) to inhibit the formation of melanocytes.^115^ Infection experiments were performed in 2 dpf zebrafish larvae unless states otherwise. At this stage, the sex differentiation is not yet complete.^116^ All procedures involving zebrafish embryos were according to local animal welfare regulations. The breeding of zebrafish in authorized institutions is in full compliance with the Dutch law on animal research. This is supervised by the local Animal Welfare Body (Instantie voor Dierenwelzijn, IvD) of the VU University. All used research protocols adhere to the international guidelines on the protection of animals used for scientific purposes, the EU Animal Protection Directive 2010/63/EU^∗^. This allows zebrafish embryos to be used up to the moment that they are able to independently take up external food (5 dpf) without additional approval by the Central Committee for Animal Experiments in the Netherlands (Centrale Commissie Dierproeven, CCD). Because embryos used in these studies meet these criteria, these specific studies are therefore approved by the IvD VU.

### Method details

#### Simultaneous total host-pathogen RNA isolation

*Casper* zebrafish larvae were injected with similar doses of the pneumolysin deficient *S*. *pneumoniae* D39V (PLY-) mutant strain or the isogenic complemented *S*. *pneumoniae* D39V (PLY+) strain at 2 dpf as described below. At 8 h post injection (hpi), 100 individually infected larvae per group were anesthetized with 0.02% buffered 3-aminobenzoic acid methyl ester (pH 7.0) (Tricaine; Sigma-Aldrich, A5040), pooled into one biological replicate. In total 3 biological replicates per group (n = 6) were isolated. To minimize transcriptional changes during sample handling, samples were snap-frozen in liquid nitrogen immediately after the study was completed. Further, we simultaneously harvested host and pathogen cells and isolated combined total RNA. To harvest the total RNA, we treated the infected larvae with a concentrated solution of ammonium sulfate to prevent any protein-dependent RNA degradation.^117^ Each milliliter of the ammonium sulfate solution (pH 5.2) contained 0.7 g (NH_4_)_2_SO_4_. The saturated solution also contained 20 mM EDTA and 25 mM sodium citrate. Three parts of saturated solution of ammonium sulfate was added directly to one part of medium. The suspension was vigorously pipetted to ensure the complete mixing of the ammonium sulfate solution and the samples, and incubated further (room temperature, 5 min). The suspension was collected and centrifuged at full speed (20 min, 4°C, 10,000 × *g*). The supernatant was removed and the cell pellet was snap-frozen with liquid nitrogen.

In a 1.5 mL screw cap tube, a PCR tube full of sterile, RNase-free glass beads (100 μm, BioSpec, Cat# 11079101) were added together with 50 μL 10% SDS and 500 μL phenol-chloroform. The frozen pellet was resuspended in TE solution (10 mM Tris-HCl, 1 mM Na_2_DTA, pH 8.0, DEPC treated mQ). The cell suspension was added into the screw cap tube and bead-beaten three times, 45 s each. Tubes were immediately placed on ice and centrifuged at full speed at 4°C to separate organic and aqueous phases. The aqueous phase was pipetted out and back-extraction was performed on the organic phase to optimize RNA yield. Phenol was further depleted from the aqueous phase by a round of chloroform extraction. After vigorous vortexing, the mixture was again centrifuged at full speed, 4°C. The aqueous phase was pipetted into a new Eppendorf tube and nucleic acids were alcohol-precipitated: 50 μL NaOAc 3M and 1 mL of cold isopropanol were added and mixed thoroughly. The mixture was incubated for at least 30 min at −20°C before pelleting by centrifugation (full speed, 4°C). Supernatant was removed gently and the nucleic acid pellet was resuspended in ice-cold 75% ethanol before re-pelleting was performed (full speed, 4°C). Ethanol washing was performed once more. The pellet was air-dried before DNase treatment.

DNase treatment was performed according to the manufacturer’s protocols (RNase-free DNase I recombinant; Roche, Cat# 4716728001) for 1 h at room temperature. To remove DNase and gDNA-derived nucleotides, phenol-chloroform extraction, chloroform extraction, isopropanol precipitation and ethanol washing were performed as previously mentioned. Total RNA was resuspended in 30 μL TE buffer. The quantity and quality of total RNA was estimated by Nanodrop and a 1% bleach gel was employed to interrogate the presence of genomic DNA and rRNA bands (23S, 2.9 kbp and 16S, 1.5 kbp).^118^

#### Library preparation and sequencing

Total RNA was isolated and the RNA quality checked using chip-based capillary electrophoresis. Samples were simultaneously depleted from eukaryotic and Gram positive ribosomal RNAs by dual rRNA-depletion as previously described.^11^ Stranded cDNA library preparation with the TruSeq® Stranded Total RNA Sample Preparation Kit (Illumina, Cat# 11079101) according to the manufacturer’s protocol. Sequencing was performed for twelve samples in one lane of Illumina NextSeq 500, High Output Flowcell in 85 single end mode. Libraries were demultiplexed and analyzed further. Raw libraries are accessible at https://www.ncbi.nlm.nih.gov/geo/with the accession number GSE123988.

#### Data analysis

Quality of raw libraries was checked^109^ (FastQC v0.11.8, Babraham Bioinformatics, UK). In order to improve the quality of alignment, we trimmed the reads using the following criteria: (i) removal of adapter sequence, if any, based on TruSeq3-SE library, (ii) removal of low quality leading and trailing nucleotides, (iii) a five-nucleotide sliding window was created for surviving reads, in which the average quality score must be above 20 and (iv) minimum remaining length must be above 50 (Trimmomatic v0.38).^108^ The quality of trimmed reads was confirmed using FastQC.^109^

We opted to align the reads to a chimeric genome containing the concatenated circular genome of *S*. *pneumoniae* onto the zebrafish genome. Here, we created chimeric genomes by concatenating the circular genome of *S*. *pneumoniae* strain D39V (GCF_003003495) onto the genome of *Danio rerio* (http://ftp.ensembl.org/pub/release-94/fasta/danio_rerio/dna/, accessed 13 November 2018). The corresponding annotation file was downloaded from the same ftp folders. Alignment was performed by RNA-STAR (v2.6.0a)^106^ with the following options: (i) alignIntronMax 1 and (ii) sjdbOverhang 80. The aligned reads were then summarized (featureCounts v1.6.3) according to the chimeric annotation file in stranded, multimapping (-M), fractionized (--fraction) and overlapping (-O) modes.^119^ The single-pass alignment was selected onto chimeric genome was selected to minimize false discovery rate.

We then analyzed host and pathogen libraries separately in R (R v3.5.2). Because rRNA depletion was successful (relative pneumococcal rRNA count ∼2.18%, relative zebrafish rRNA count ∼0.4%), we did not exclude reads which aligned to genes encoding ribosomal RNA in subsequent analysis. Differential gene analysis was performed by DESeq2 v1.22.1 and genome-wide fold change was calculated between the transcriptional response of the two bacterial strains, PLY+ and PLY-.^38^ Value of fold change was set to zero if the corresponding *q* value is reported to be NA. Enrichment tests for functional analysis were performed by the built-in function, *fisher*.*test()*.

Soft clustering was performed on the normalized centered gene expression values with a fuzzifier value of 1.5 to obtain a better view of the dynamics of gene expression during infection.^42^ Functional enrichment analysis was performed using WEB-based GEne Set AnaLysis Toolkit (WebGestalt).^41^ For Gene Ontology (GO) enrichment analysis at least 3 genes were required and p value < 0.05. Gene Set Enrichment Analysis was analyzed by Webgestalt 2019.

#### RT-qPCR confirmation of host genes

To obtain total RNA, we first anesthetized zebrafish larvae (n = 50 per group unless stated otherwise) in 0.02% Tricaine (Sigma-Aldrich, Cat# A5040) in egg water at 8 hpi and collected them in a 2 mL Eppendorf tube. Then, excess egg water was removed and 350 μL lysis buffer added (NucleoSpin; MACHEREY-NAGEL, Cat# 740955.50). The larvae were homogenized by repeatedly drawing through a 26-gauge needle into a syringe and expelled. Isolation of total RNA was performed using NucleoSpin RNA isolation kit (NucleoSpin, MACHEREY-NAGEL, Cat# 740955.50). cDNA was synthesized from total RNA by SuperScript® IV VILO Master Mix (Invitrogen, Cat# 11756050). Primers were designed across exons (for host genes) while retaining its species specificity as confirmed by *in silico* PCR on the opposite species (*i*.*e*., host primers to *S*. *pneumoniae* genome, *vice versa*) (Table S4). Amplification efficiency for each primer was calculated based on primer ability to double amount of product per cycle. The qPCR mix contained forward and reverse primers, with either PowerTrack™ SYBR Green Master Mix (Applied Biosciences; Cat# A46012) or SsoAdvanced Universal SYBR Green Supermix (Bio-Rad; Cat# 172–5270), and cDNA. The reactions were on triplicates and two different cDNA concentrations. Subsequently, PowerTrack™ SYBR Green Master Mix and primers for the selected genes (Table S4) were added and RT-qPCR was performed on a StepOnePlus™ Real-Time PCR System (Applied Biosystems) or a CFX96 Touch Real-Time PCR Detection System (Bio-Rad). As reference genes we used the *Mobk13 (mob4)* for zebrafish genes and *gyrA* for pneumococcal genes.^11^^,^^120^ The RT-qPCR fold change was calculated by the ΔΔCt method.^107^

#### Fluorescence imaging of zebrafish larvae

Zebrafish larvae infected with the *S*. *pneumoniae* D39V SsbB-reporter strain were collected at 8 hpi and fixated overnight in 4% paraformaldehyde in PBS, washed with PBS, prior to confocal imaging. Confocal images were generated with a Rescan Confocal Microscope 2 (RCM2, Confocal.nl) with a Hamamatsu ORCA flash 4.0 v3. For optimal imaging, larvae were embedded in 1.5% low-melting-point agarose dissolved in PBS in an open uncoated 8-well microscopy μ-Slide (http://ibidi.com). ImageJ software was used to process the confocal images, specifically for brightness/contrast enhancements as well as for creating merged images.^121^ Figures were made using Adobe Illustrator and Adobe InDesign.

#### Time-lapse fluorescence imaging of zebrafish larvae

Screening and imaging of *Tg(cxcl18b*:*eGFP)* or *Tg(mpx*:*GFP)* zebrafish larvae were performed with a Zeiss Axio Zoom V16 stereo microscope with a Hamamatsu ORCA flash 4.0 camera attached. Time-lapse confocal images were acquired with a Nikon A1R confocal microscope. *Tg(cxcl18b*:*eGFP)* zebrafish larvae were imaged 1 h after injection into the hindbrain ventricle with 2,000 CFU of *S*. *pneumoniae* PLY+, *S*. *pneumoniae* PLY- or PBS. Images were obtained at 1 h intervals for 20 h. *Tg(mpx*:*GFP) casper* zebrafish larvae were imaged 1 hpi in the hindbrain ventricle with 2,000 CFU of mCherry labeled *S*. *pneumoniae* D39V and images were obtained at 30 min intervals for 9 h. After injection, the anesthetized larvae were embedded in 1.5% low-melting point agarose dissolved in egg water dissolved in egg water (60 μg/mL sea salts (Sigma-Aldrich, S9883) in MiliQ) with 0.02% (w/v) Tricaine (Sigma-Aldrich, Cat# A5040) and GSK′872 (RIPK3 inhibitor) (MedChemExpress; Cat# HY-101872) in 1% DMSO was added to a final concentration of 100 μM or 1% DMSO alone (vehicle control) in an open uncoated 8-well chamber slide (Ibidi, Cat# 80826) and kept at 28°C in a temperature-controlled chamber (Okolab). NIS Elements and ImageJ were used for image analysis and neutrophil quantification.

#### Zebrafish survival experiments

Pneumolysin deficient *S*. *pneumoniae* D39V PLY- mutant strain or complemented *S*. *pneumoniae* D39V PLY+ strain were grown in C + Y medium until mid-log phase (OD595nm–0.2), harvested by centrifugation (6,000 RPM for 10 min), washed with sterile PBS, harvested again by centrifugation, and finally resuspended in 0.125% (w/v) phenol red solution (Sigma-Aldrich; P0290) to aid visualization of the injection process. Prior to injection, 2 days post fertilization (dpf) larvae were mechanically dechorionated if necessary and anesthetized in 0.02% (w/v) Tricaine (Sigma-Aldrich, Cat# A5040). Larvae were randomly assigned to experimental groups. The bacterial suspension was then injected into the hindbrain ventricle of *casper* zebrafish larvae at 2 dpf. After injection, the larvae were kept in 6-well plates containing egg water (60 μg/mL sea salts (Sigma-Aldrich; S9883) in demi water) at 28°C and the mortality rate monitored at fixed time-points as described previously.^26^ To assess the effect of necroptosis pathway inhibition on survival, infected zebrafish larvae were treated orally with 100 μM GSK′872 (RIPK3 inhibitor) (MedChemExpress; HY-101872) in 1% DMSO, or 1% DMSO alone (vehicle control) by adding the compound to egg water for the whole infection course with refreshment of the egg water and compound every 24 h. All experiments were performed in triplicate. Survival graphs were generated with GraphPad Prism 8.0 and analyzed with the log rank (Mantel-Cox) test. Results were considered significant at p values < 0.05.

#### Bacterial load and competitive index

To determine the bacterial load at different time-points, infected zebrafish larvae were anesthetized in 0.02% Tricaine (Sigma-Aldrich, Cat# A5040) in egg water, transferred to a 1.5 mL screwcap tube (1 larva per tube) filled with 1.0 mm glass beads (Sigma-Aldrich, Cat# Z250473) to ∼25% capacity of the tubes’ volume, placed in a microvial rack, and violently shaken (3 times 10 s, 10 s interval) in a bead beater (BioSpec Products; Mini Beadbeater) to disrupt the cells and tissues. Subsequently, serial dilution plating was performed on Columbia Blood Agar (BD Biosciences, Cat# 211124) plates supplemented with 5% defibrinated sheep blood (bioTRADING, Cat# BTSG100) (COS plates), 10 mg/L colistin sulfate and 5 mg/L oxolinic acid (COBA; Oxoid, Cat# SR0126), to inhibit growth of commensal bacteria in zebrafish. The plates were incubated O/N at 37°C and quantified the next day. To determine the competitive indexes, zebrafish larvae were co-injected at 2 dpf with two pneumococcal strains of interest mixed in a 1:1 ratio into the hindbrain ventricle. The number of CFU per injection was determined by quantitative plating of the injection volume on COS plates containing appropriate antibiotics. At 24 hpi, anesthetized larvae were homogenized as described above and serial dilution plating was performed on COS plates containing COBA supplement and appropriate antibiotics, to quantify the number of both pneumococcal strains in each larva. The competitive indexes were then calculated by dividing the ratio of the test strain divided by the reference strain. All experiments were performed in duplicate. Scatterplot and competitive index plots were generated with GraphPad Prism 8.0 and analyzed with unpaired t test or one sample t test respectively.

### Quantification and statistical analysis

All survival data were analyzed by log rank test (Mantel-Cox). All data were analyzed by Student’s t-test for comparison of two groups or two-way ANOVA followed by Sidak’s or Tukey’s post hoc test. Bacterial load and competitive index data were analyzed by unpaired t test or one sample t test respectively. The number of subjects and biological replicates is indicated in the figure legends. All data including error bars are presented as mean ± SD or mean ± SEM. p values < 0.05 and *q* values < 0.05 were considered statistically significant, reported as ∗p value < 0.05 in the figure legends, unless stated otherwise. All statistical analyses were performed using GraphPad Prism 8.0.

### Additional resources

The dual RNA-seq data is easily accessible, searchable, and downloadable via a web-based platform: https://veeninglab.com/dual-danio.

## Data Availability

Dual RNA-seq data have been deposited at GEO and are publicly available as of the date of publication. Accession numbers are listed in the key resources table. The DOI is listed in the key resources table. This paper does not report original code. Any additional information required to reanalyze the data reported in this paper is available from the lead contact upon request.

## Supplemental information

Document S1. Figures S1–S4 and Table S4

Table S1. *Danio rerio* differential gene expression

Table S2. Streptococcus pneumoniae D39V differential gene expression

Table S3. Functional enrichment analysis of differentially expressed *Danio rerio* genes

Document S2 Article plus supplemental information
