# Supplementary material for: Pneumolysin promotes host cell necroptosis and bacterial competence during pneumococcal meningitis as shown by whole-animal dual RNA-seq
Source: Cell Rep. 2022 Dec 20;41(12):111851. doi: 10.1016/j.celrep.2022.111851 (PMC9794515; doi:10.1016/j.celrep.2022.111851)
Supplement: Document S1. Figures S1–S4 and Table S4 [file mmc1.pdf]

Cell Reports, Volume *41*

Supplemental information

Pneumolysin promotes host cell necroptosis and bacterial competence during pneumococcal meningitis as shown by whole-animal dual RNA-seq

Kin Ki Jim, Rieza Aprianto, Rutger Koning, Arnau Domenech, Jun Kurushima, Diederik van de Beek, Christina M.J.E. Vandenbroucke-Grauls, Wilbert Bitter, and Jan-Willem Veening

SUPPLEMENTAL FIGURES

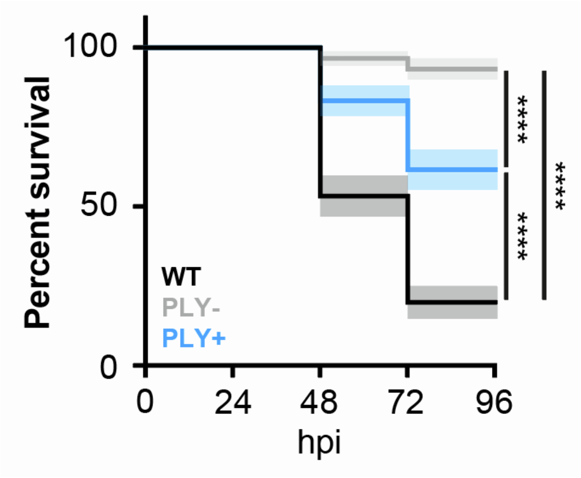

Figure S1. Complementation of the pneumolysin gene partially restores virulence of the pneumolysin-deficient *S. pneumoniae* PLY- strain in a zebrafish larval pneumococcal meningitis model. Survival curves of 2 dpf zebrafish larvae injected with *S. pneumoniae* D39V wildtype, *S. pneumoniae* D39V PLY+ or *S. pneumoniae* D39V PLY- strain in the hindbrain ventricle. Larvae were infected with 300 CFU. The data represent the mean ± SEM of three biological replicates with 20 embryos in each group; ****p value <0.0001; determined by log-rank test.

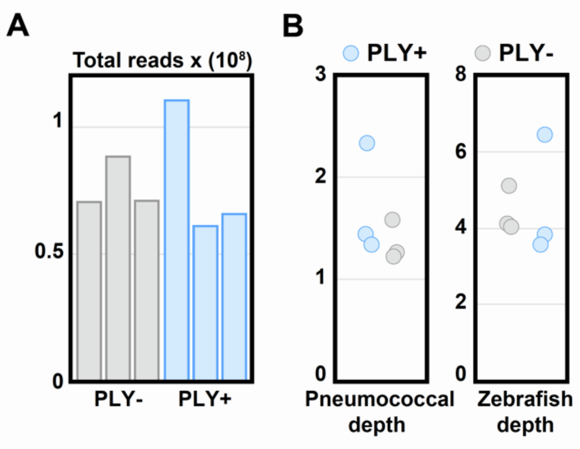

Figure S2. Total reads and depth. (A) On average, each library has 77 million reads (467 million reads in total); equivalent to 6.2 billion nucleotides. **(B)** Average depth of pneumococcal reads is 1.54 and zebrafish depth is 4.54.

	Danio rerio	*S. pneumoniae*
Total Genes	25,592	2,133
- Unexpressed	3,462	201
- *q* value > 0.05	15,727	-
- *q* value > 0.5	-	1.924
Working Libraries	**6,403**	**8**
	**(25%)**	**(0.4%)**
	vs total genes	vs total genes

Figure S3. Zebrafish and pneumococcal working libraries. Two gene fractions were removed to simplify downstream analysis. After removal of unexpressed genes and non-significant genes the zebrafish working library contained 6,403 genes (25% of *D. rerio* genes) whereas the pneumococcal working library contained 8 genes (0.4% of *S. pneumoniae* genes).

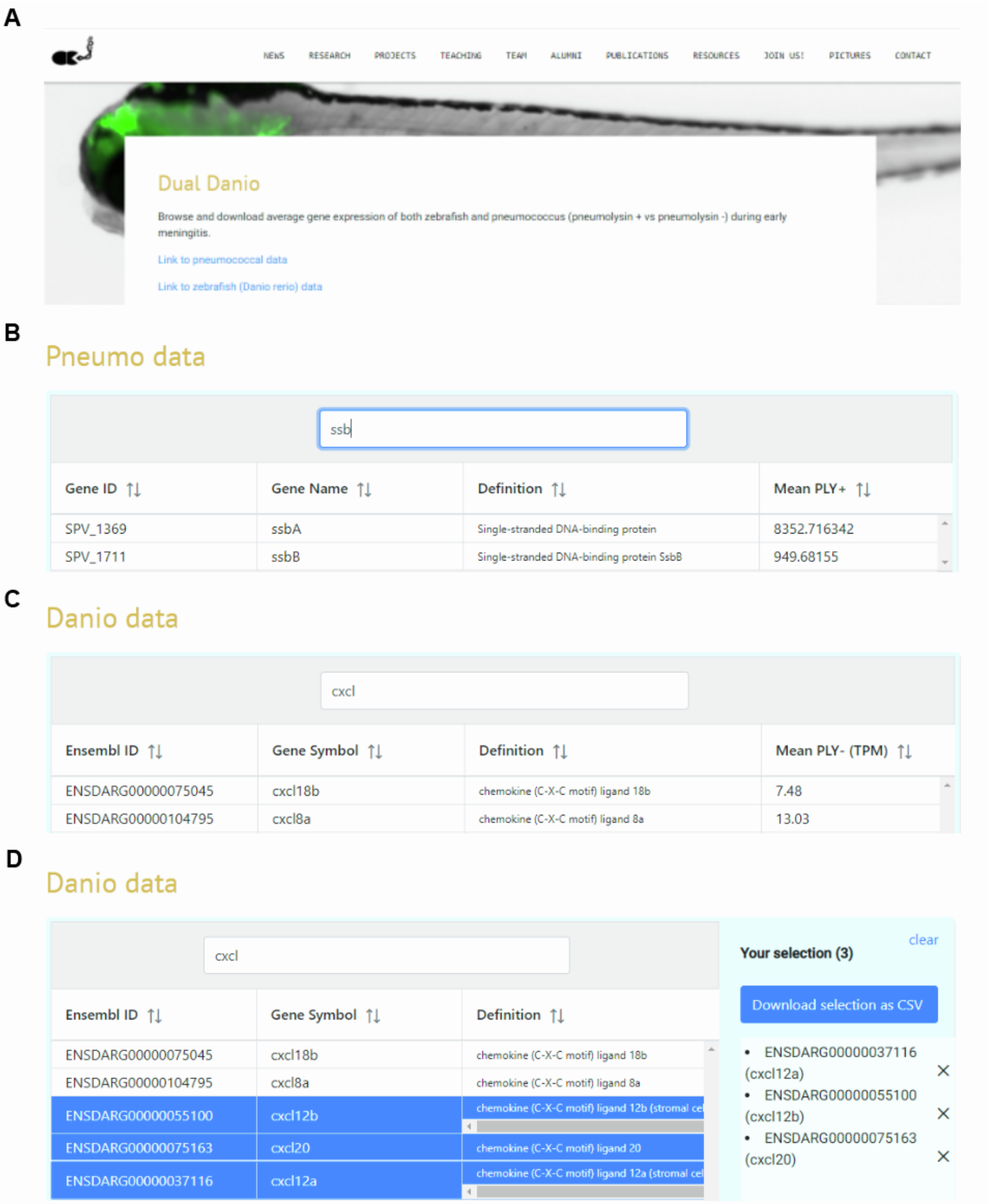

Figure S4. Easy access of the complete dual RNA-seq database. (A) Screenshot of the web-based platform with search result example summary for *D. rerio* **(B)** or *S. pneumoniae* **(C)***.* The RNA-seq data of a single gene or multiple genes can be easily exported **(D)**.

SUPPLEMENTARY TABLES

Table S4. Primers

Oligonucleotide name	Sequence (5' to 3')	Description
LK162	CTAGCCTTGACAACTAGCCAATC	Construction of *ply*::cam^r
LK163	CTCACAAAAATCCGAGCTCCACCGCTTCTACCTCCTAATAAGTTCCTGG	Construction of *ply*::cam^r
LK164	CCAGGAACTTATTAGGAGGTAGAAGCGGTGGAGCTCGGATTTTTGTGAG	Construction of *ply*::cam^r
LK165	CGCAAGCATTCTCCTCTCCGCTAGGGCGCTGGCAAG	Construction of *ply*::cam^r
LK166	CTTGCCAGCGCCCTAGCGGAGAGGAGAATGCTTGCG	Construction of *ply*::cam^r
LK167	TGCAAATAGAAAGTTTCAGCC	Construction of *ply*::cam^r
LK283	GCCCGGGATCCTCGCAGGGAGTCTAATAAG	Construction of *ply*::cam^r complemented
LK284	CTTTATTTGCCATCTTCTACCTCCTTTCATTTACTTTGATAACCATAGTATAAC	Construction of *ply*::cam^r complemented
LK285	GTTATACTATGGTTATCAAAGTAAATGAAAGGAGGTAGAAGATGGCAAATAAAG	Construction of *ply*::cam^r complemented
LK286	GCCCGTCTAGAGACTAGTACACTGGCACTTC	Construction of *ply*::cam^r complemented
OVL2549	GTGATGCGTCTCGTCTTGGATTTTTGTGAGCTTGGA	Construction of *comCDE*::ery^r
OVL2771	GTGATGCGTCTCGAGGTACCCGAGCGGC	Construction of *comCDE*::ery^r
OVL506	ATTCATAGGCGCGTGCTTCTTC	Construction of *comCDE*::ery^r
OVL2548	GTGATGCGTCTCGAAGAAAAAGCCGGGAAAA	Construction of *comCDE*::ery^r
OVL2733	CGCCGCCGCCTGATGAGCCGCCATTCTTATTGTTTTTGTAAAC	Construction of *comCDE*::ery^r
OVL1667	GTAAATAGAGCTAGTATGAC	Construction of *comCDE*::ery^r
OVL2772	GTGATGCGTCTCGAGGTACCCGTTACGACGCG	Construction of hlpA-GFP::trmp^r and hlpA_hlpA-mCherry::trmp^r
OVL43	AACAAGTCAGCCACCTGTAG	Construction of hlpA-GFP::trmp^r and hlpA_hlpA-mCherry::trmp^r
OVL2769	GTGATGCGTCTCGAAGAATATGCCTCCTACTAGTG	Construction of hlpA-GFP::trmp^r and hlpA_hlpA-mCherry::trmp^r

OVL2770	GTGATGCGTCTCGACCATTAAAAAGCCTATTGTATC	Construction of hlpA-GFP::trmp^r and hlpA_hlpA-mCherry::trmp^r
OVL46	CGTGGCTGACGATAATGAGG	Construction of hlpA-GFP::trmp^r and hlpA_hlpA-mCherry::trmp^r
ADP1/45	GATAAAGGCGTAGGAGACACTGACC	Construction of *cbpD*::spec^r
ADP1/46+SphI	CGATGCATGCGTGTTAAACTTCGACCGCACCTG	Construction of *cbpD*::spec^r
ADP1/47+HindIII	CGATAAGCTTAATATAAAGCGATGATTGCCTTC	Construction of *cbpD*::spec^r
ADP1/48	CAACTTATCACGGGAACTGGTCGAG	Construction of *cbpD*::spec^r
sPG11+SphI	CGATGCATGCAGGAGGCATATCAAATGAAC	Construction of *cbpD*::spec^r
sPG12+HindIII	CGATAAGCTTTTATAAAAGCCAGTCATTAG	Construction of *cbpD*::spec^r
col1a1b_FW	TGGACAGCCTGGTGCTAAAG	RT-qPCR
col1a1b_RV	CAGCAGGTCCCTGTGGTC	RT-qPCR
pdxp_FW	CTTGTGGGCTACGACGAGAG	RT-qPCR
pdxp_RV	CCAGAACCCGGGGTGATCC	RT-qPCR
nme2b.2_FW	TTGAAGTCGGCAGGAACCTGAT	RT-qPCR
nme2b.2_RV	GTTCTGGAAGTGTCTCTGTTCAC	RT-qPCR
ccl25b_FW	CAGCATCGCACAAGGTTACT	RT-qPCR
ccl25b_RV	TCCGTCTGTTAGCTGCACTC	RT-qPCR
cxcl18b_FW	GCAGGGAGAAGTTCTGGCTAA	RT-qPCR
cxcl18b_RV	ACTTTGTCGCAGTTTGGTCT	RT-qPCR
mob4_FW	GCTCAATGGCTTGGCAGTTA	RT-qPCR
mob4_RV	AATCAATGGCAGGGCACTCT	RT-qPCR
ripk3_FW	GCAGTTACAAACCCAGCAAAG	RT-qPCR
ripk3_RV	GGCTCCTTCCCAGTGATAATG	RT-qPCR
